# 2-Phenyl-1*H*-imidazole

**DOI:** 10.1107/S160053681104699X

**Published:** 2011-11-09

**Authors:** Maryam Mehdizadeh Barforoush, Soheila Naderi, Ali Reza Ghanbarpour, Alireza Azhdari Tehrani, Hamid Reza Khavasi

**Affiliations:** a Department of Chemistry, Shahid Beheshti University, G. C., Evin, Tehran 1983963113, Iran

## Abstract

In the title compound, C_9_H_8_N_2_, a mirror plane lies perpendicular to the phenyl and imidazole rings and passes through the bridging C—C bond, so that the imidazole ring is disordered over two sites about the mirror plane with the equal site occupancy; the asymmetric unit contains one half-mol­ecule. In the crystal, adjacent mol­ecules are linked *via* N—H⋯N hydrogen bonds.

## Related literature

For structures of 2-phenyl-1*H*-imidazolium salts, see: Xia *et al.* (2009 ▶); Xia & Yao (2010 ▶).
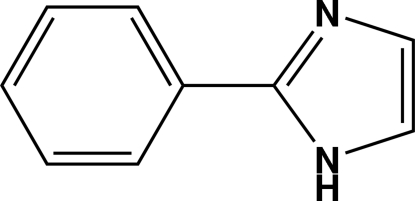


## Experimental

### 

#### Crystal data

C_9_H_8_N_2_
*M* *_r_* = 144.17
Orthorhombic, 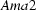

*a* = 10.0740 (15) Å
*b* = 18.151 (4) Å
*c* = 4.1562 (10) Å
*V* = 760.0 (3) Å^3^
*Z* = 4
Mo *K*α radiation
μ = 0.08 mm^−1^
*T* = 298 K
0.17 × 0.12 × 0.10 mm

#### Data collection

Stoe IPDS 2T diffractometer
1776 measured reflections
609 independent reflections
304 reflections with *I* > 2σ(*I*)
*R*_int_ = 0.082

#### Refinement

*R*[*F* ^2^ > 2σ(*F* ^2^)] = 0.056
*wR*(*F* ^2^) = 0.095
*S* = 0.98
609 reflections
56 parameters
1 restraint
H-atom parameters constrained
Δρ_max_ = 0.14 e Å^−3^
Δρ_min_ = −0.09 e Å^−3^

### 

Data collection: *X-AREA* (Stoe & Cie, 2002 ▶); cell refinement: *X-AREA*; data reduction: *X-RED* (Stoe & Cie, 2002 ▶); program(s) used to solve structure: *SHELXS97* (Sheldrick, 2008 ▶); program(s) used to refine structure: *SHELXL97* (Sheldrick, 2008 ▶); molecular graphics: *ORTEP-3 for Windows* (Farrugia, 1997 ▶); software used to prepare material for publication: *WinGX* (Farrugia, 1999 ▶).

## Supplementary Material

Crystal structure: contains datablock(s) global, I. DOI: 10.1107/S160053681104699X/xu5367sup1.cif

Structure factors: contains datablock(s) I. DOI: 10.1107/S160053681104699X/xu5367Isup2.hkl

Supplementary material file. DOI: 10.1107/S160053681104699X/xu5367Isup3.cml

Additional supplementary materials:  crystallographic information; 3D view; checkCIF report

## Figures and Tables

**Table 1:** Hydrogen-bond geometry (Å, °)

*D*—H⋯*A*	*D*—H	H⋯*A*	*D*⋯*A*	*D*—H⋯*A*
N1—H1*B*⋯N1^i^	0.86	2.05	2.891 (3)	165

Symmetry code: (i) 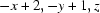.

**Fig. 1.**
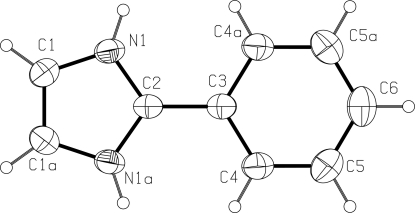
The molecular structure with the atom-numbering scheme. Displacement ellipsoids are drawn at 30% probability level.

**Fig. 2.**
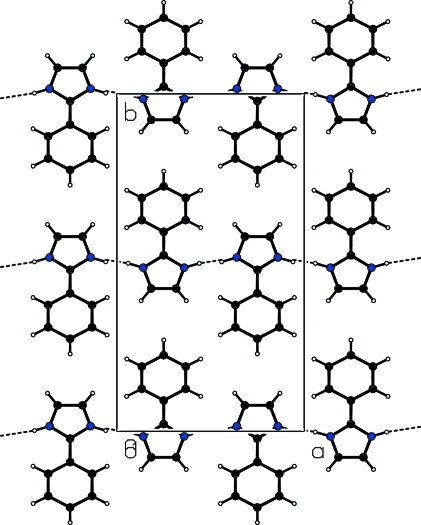
Packing diagram.

## supplementary crystallographic information

### Comment

2-Phenylimidazole, as an important compound with potential N donor atom that may contribute in classical hydrogen bonding in generation of supramolecular assemblies. There are some crystal structure reports that show 2-phenylimidazole can be protonated (Xia *et al.*, 2009; Xia & Yao, 2010).

The asymmetric unit of the title compound contains one half-molecule, a mirror plane passes through the C—C connecting two rings (Fig. 1). In this molecule the bond lengths and angles are within normal ranges. The imidazole and phenyl rings are nearly co-planar. The intermolecular N—H···N hydrogen bonds (Table 1) occurs in the crystal structure (Table 1).

### Experimental

The title compound has been obtained during the stirring of 2-phenyl-1*H*-imidazole and aniline in 1:1 molar ration in methanol for synthesis of co-crystal of reagents. The suitable crystals for X-ray analysis were obtained by slow evaporation from methanol solution after one week (yield; 86.5%).

### Refinement

All of the H atoms were positioned geometrically with C—H = 0.93 and N—H = 0.86 Å, and constrained to ride on their parent atoms, with *U*_iso_(H) = 1.2*U*_eq_(C,N). The molecule is disordered over two sites in the crystal structure and H1B atom is in 50% occupancy. Friedel pairs were merged as no significant anomalous scatterings.

### Crystal data

**Table:**

C_9_H_8_N_2_	*F*(000) = 304
*M**_r_* = 144.17	*D*_x_ = 1.26 Mg m^−^^3^
Orthorhombic, *A**m**a*2	Mo *K*α radiation, λ = 0.71073 Å
Hall symbol: A 2 -2a	Cell parameters from 1776 reflections
*a* = 10.0740 (15) Å	θ = 3.0–29.1°
*b* = 18.151 (4) Å	µ = 0.08 mm^−^^1^
*c* = 4.1562 (10) Å	*T* = 298 K
*V* = 760.0 (3) Å^3^	Prism, colorless
*Z* = 4	0.17 × 0.12 × 0.10 mm

### Data collection

**Table:**

Stoe IPDS 2T diffractometer	*R*_int_ = 0.082
graphite	θ_max_ = 29.1°, θ_min_ = 3.0°
rotation method scans	*h* = −13→11
1776 measured reflections	*k* = −19→24
609 independent reflections	*l* = −5→4
304 reflections with *I* > 2σ(*I*)	

### Refinement

**Table:**

Refinement on *F*^2^	Secondary atom site location: difference Fourier map
Least-squares matrix: full	Hydrogen site location: inferred from neighbouring sites
*R*[*F*^2^ > 2σ(*F*^2^)] = 0.056	H-atom parameters constrained
*wR*(*F*^2^) = 0.095	*w* = 1/[σ^2^(*F*_o_^2^) + (0.0288*P*)^2^] where *P* = (*F*_o_^2^ + 2*F*_c_^2^)/3
*S* = 0.98	(Δ/σ)_max_ = 0.001
609 reflections	Δρ_max_ = 0.14 e Å^−^^3^
56 parameters	Δρ_min_ = −0.09 e Å^−^^3^
1 restraint	Extinction correction: *SHELXL97* (Sheldrick, 2008)
Primary atom site location: structure-invariant direct methods	Extinction coefficient: 0.009 (3)

### Special details

**Table:**

Geometry. All e.s.d.'s (except the e.s.d. in the dihedral angle between two l.s. planes) are estimated using the full covariance matrix. The cell e.s.d.'s are taken into account individually in the estimation of e.s.d.'s in distances, angles and torsion angles; correlations between e.s.d.'s in cell parameters are only used when they are defined by crystal symmetry. An approximate (isotropic) treatment of cell e.s.d.'s is used for estimating e.s.d.'s involving l.s. planes.
Refinement. Refinement of *F*^2^ against ALL reflections. The weighted *R*-factor *wR* and goodness of fit *S* are based on *F*^2^, conventional *R*-factors *R* are based on *F*, with *F* set to zero for negative *F*^2^. The threshold expression of *F*^2^ > σ(*F*^2^) is used only for calculating *R*-factors(gt) *etc*. and is not relevant to the choice of reflections for refinement. *R*-factors based on *F*^2^ are statistically about twice as large as those based on *F*, and *R*- factors based on ALL data will be even larger.

### Fractional atomic coordinates and isotropic or equivalent isotropic displacement parameters (Å^2^)

**Table:**

	*x*	*y*	*z*	*U*_iso_*/*U*_eq_	Occ. (<1)
C1	0.8165 (3)	0.57694 (18)	0.2820 (10)	0.0683 (10)	
H1	0.8705	0.6129	0.1911	0.082*	
C2	0.75	0.4784 (3)	0.5302 (10)	0.0489 (13)	
C3	0.75	0.4087 (3)	0.7033 (11)	0.0484 (12)	
C4	0.6328 (3)	0.3743 (2)	0.7893 (9)	0.0650 (9)	
H4	0.5523	0.3965	0.7387	0.078*	
C5	0.6329 (4)	0.3080 (2)	0.9474 (10)	0.0778 (12)	
H5	0.5528	0.2858	1.0015	0.093*	
C6	0.75	0.2743 (3)	1.0264 (15)	0.0799 (17)	
H6	0.75	0.2292	1.1323	0.096*	
N1	0.8591 (2)	0.51510 (13)	0.4384 (6)	0.0592 (8)	
H1B	0.9402	0.5022	0.4716	0.071*	0.5

### Atomic displacement parameters (Å^2^)

**Table:**

	*U*^11^	*U*^22^	*U*^33^	*U*^12^	*U*^13^	*U*^23^
C1	0.0563 (17)	0.0625 (19)	0.086 (3)	−0.0063 (15)	0.0075 (18)	0.007 (2)
C2	0.038 (3)	0.054 (3)	0.055 (4)	0	0	−0.010 (3)
C3	0.044 (3)	0.047 (2)	0.054 (3)	0	0	−0.014 (3)
C4	0.0494 (18)	0.069 (2)	0.077 (2)	−0.0027 (18)	−0.001 (2)	0.000 (2)
C5	0.080 (2)	0.071 (2)	0.082 (3)	−0.018 (2)	0.002 (2)	0.003 (3)
C6	0.111 (5)	0.053 (3)	0.076 (4)	0	0	0.001 (3)
N1	0.0398 (15)	0.0607 (16)	0.0771 (18)	−0.0022 (14)	0.0056 (15)	−0.0011 (19)

### Geometric parameters (Å, °)

**Table:**

C1—C1^i^	1.339 (6)	C4—C5	1.370 (5)
C1—N1	1.367 (4)	C4—H4	0.93
C1—H1	0.93	C5—C6	1.369 (4)
C2—N1^i^	1.341 (3)	C5—H5	0.93
C2—N1	1.341 (3)	C6—C5^i^	1.369 (4)
C2—C3	1.456 (6)	C6—H6	0.93
C3—C4^i^	1.383 (4)	N1—H1B	0.86
C3—C4	1.383 (4)		
			
C1^i^—C1—N1	108.34 (16)	C3—C4—H4	119.4
C1^i^—C1—H1	125.8	C6—C5—C4	120.6 (4)
N1—C1—H1	125.8	C6—C5—H5	119.7
N1^i^—C2—N1	110.2 (4)	C4—C5—H5	119.7
N1^i^—C2—C3	124.9 (2)	C5—C6—C5^i^	119.0 (5)
N1—C2—C3	124.9 (2)	C5—C6—H6	120.5
C4^i^—C3—C4	117.3 (4)	C5^i^—C6—H6	120.5
C4^i^—C3—C2	121.4 (2)	C2—N1—C1	106.6 (3)
C4—C3—C2	121.4 (2)	C2—N1—H1B	126.7
C5—C4—C3	121.3 (4)	C1—N1—H1B	126.7
C5—C4—H4	119.4		
			
N1^i^—C2—C3—C4^i^	180.0 (4)	C3—C4—C5—C6	0.4 (6)
N1—C2—C3—C4^i^	0.2 (6)	C4—C5—C6—C5^i^	0.5 (8)
N1^i^—C2—C3—C4	−0.2 (6)	N1^i^—C2—N1—C1	−0.3 (5)
N1—C2—C3—C4	−180.0 (4)	C3—C2—N1—C1	179.5 (4)
C4^i^—C3—C4—C5	−1.3 (6)	C1^i^—C1—N1—C2	0.2 (3)
C2—C3—C4—C5	178.9 (4)		

Symmetry codes: (i) −*x*+3/2, *y*, *z*.

### Hydrogen-bond geometry (Å, °)

**Table:**

*D*—H···*A*	*D*—H	H···*A*	*D*···*A*	*D*—H···*A*
N1—H1B···N1^ii^	0.86	2.05	2.891 (3)	165

Symmetry codes: (ii) −*x*+2, −*y*+1, *z*.
